# *Saccharopolyspora* sp. NFXS83 in Marine Biotechnological Applications: From Microalgae Growth Promotion to the Production of Secondary Metabolites

**DOI:** 10.3390/microorganisms11040902

**Published:** 2023-03-30

**Authors:** Constança D. F. Bertrand, Rodrigo Martins, Francisco Quintas-Nunes, Pedro Reynolds-Brandão, Maria T. B. Crespo, Francisco X. Nascimento

**Affiliations:** 1 iBET—Instituto de Biologia Experimental e Tecnológica, 2781-901 Oeiras, Portugal; constanca.bertrand@ibet.pt (C.D.F.B.); rodrigo.martins@ibet.pt (R.M.); francisco.nunes@ibet.pt (F.Q.-N.); pedro.brandao@ibet.pt (P.R.-B.); tcrespo@ibet.pt (M.T.B.C.); 2 ITQB—Instituto de Tecnologia Química e Biológica António Xavier, Universidade Nova de Lisboa, 2780-157 Oeiras, Portugal

**Keywords:** *Saccharopolyspora*, marine, microalgae, secondary metabolites, biotechnology

## Abstract

Marine bacteria are a significant source of bioactive compounds for various biotechnological applications. Among these, actinomycetes have been found to produce a wide range of secondary metabolites of interest. *Saccharopolyspora* is one of the genera of actinomycetes that has been recognized as a potential source of these compounds. This study reports the characterization and genomic analysis of *Saccharopolyspora* sp. NFXS83, a marine bacterium isolated from seawater from the Sado estuary in Portugal. The NFXS83 strain produced multiple functional and stable extracellular enzymes under high-salt conditions, showed the ability to synthesize auxins such as indole-3-acetic acid, and produced diffusible secondary metabolites capable of inhibiting the growth of *Staphylococcus aureus*. Furthermore, when *Phaeodactylum tricornutum* was co-cultivated with strain NFXS83 a significant increase in microalgae cell count, cell size, auto-fluorescence, and fucoxanthin content was observed. Detailed analysis revealed the presence of clusters involved in the production of various secondary metabolites, including extracellular enzymes, antimicrobial compounds, terpenes, and carotenoids in the genome of strain NFXS83. Ultimately, these findings indicate that *Saccharopolyspora* sp. NFXS83 has a significant potential for a wide range of marine biotechnological applications.

## 1. Introduction

Marine environments harbor diverse and rich bacterial populations, which play key roles in several aspects of marine ecology and global nutrient cycles [1]. Amongst marine microorganisms, actinobacteria are of special interest due to their relevant impacts in marine ecosystems and increased ability to synthesize a wide variety of secondary metabolites and bioactive compounds of biotechnological interest, including antibiotics, antitumoral agents, pigments, and enzymes [2,3,4,5]. Marine actinobacteria are found in several marine environments, including surface waters [6], sediments [7,8], and even adhered to the surfaces of other marine organisms, such as macroalgae [9] and marine invertebrates [10]. These bacteria play a role in the cycling of nutrients in marine ecosystems and can interact with other marine organisms in a wide range of trophic interactions. For example, some actinobacteria have been shown to produce compounds that inhibit the growth of harmful algae, potentially affecting their abundance and distribution in the ocean [11]. On the other hand, some actinobacteria producing antimicrobial compounds may have a mutualistic relationship with marine organisms such as corals, protecting these organisms from pathogens and other predators [12].

The stressful nature of marine environments (e.g., high salinity, fluctuating temperatures and light intensity, low nutrient concentrations, competition) greatly impacts their associated microorganisms, including marine actinobacteria. This leads to strong adaptations (genetic and phenotypic) to stress conditions by these microorganisms, favoring the biosynthesis of unique bioactive compounds [13]. Recent studies have revealed the biotechnological properties of some marine actinobacteria [5], however, due to their increased genetic and phenotypic diversity much of their biotechnological potential is still untapped.

*Saccharopolyspora* are gram-positive, aerobic, non-motile actinobacteria that are largely distributed throughout the terrestrial and marine environments with half of their species described as halophilic or halotolerant [14]. Moreover, *Saccharopolyspora* are one among the various genera of actinomycetes recognized as a potential source of novel bioactive compounds [14]. For example, some species of *Saccharopolyspora* are known to produce several antibiotics, including erythromycin [15,16].

In this work, the marine actinomycete *Saccharopolyspora* sp. NFXS83, a bacterium isolated from the seawater surface (photic zone) in the Sado estuary, Portugal, is characterized in detail and its genome sequence is analyzed and discussed. The results obtained herein bring new insights into the role of *Saccharopolyspora* in marine environments and their potential for use in a wide range of biotechnological applications, including the ability to promote microalgae growth and their accumulation of valuable compounds, and the production of several secondary metabolites of relevance.

## 2. Materials and Methods

### 2.1. Isolation and Identification of Strain NFXS83

Strain NFXS83 was isolated as part of an effort to characterize the microalgae and bacterial communities of Portuguese marine waters. For this, seawater was collected and used for the isolation of microorganisms. The surface seawater from the photic zone was collected from the Sado estuary, Portugal, in June 2021 and immediately transported to the lab. The water was filtered using sterile cellulose filters (5 μm) and used for the isolation of bacteria by spreading 50 mL of filtered seawater in Marine Agar (MA, Condalab, Spain) plates. The plates were incubated at 26 °C in the dark for 12 days. After the incubation period, individual colonies were selected and streaked until pure cultures were obtained. Strain NFXS83 was isolated, maintained in MA plates and grown in Marine Broth (MB, Condalab, Spain) whenever necessary. The strain was kept at glycerol stocks at −80 °C until further use.

The strain NFXS83 16S rRNA gene sequencing was conducted following genomic DNA extraction from an overnight culture (in MB) using the PureLink™ Genomic DNA kit (Invitrogen, Waltham, MA, USA) according to the manufacturer’s instructions. The obtained DNA was analyzed for its purity and integrity using a Nanodrop^®^ (ND-1000, Thermo Scientific, Waltham, MA, USA) and was used for the PCR amplification reaction. The 16S rRNA gene was amplified using primers 27F and 1492R following the conditions described elsewhere [17]. The near complete 16S rRNA sequence of strain NFXS83 was obtained following its sequencing, which was performed using an external service provided by Eurofins Genomics (Germany).

*Saccharopolyspora* type strains and other related bacteria 16S rRNA genes were obtained from the NCBI database (https://www.ncbi.nlm.nih.gov/ accessed on February 2023) and aligned using MUSCLE [18]. The 16S rRNA-based phylogenetic analysis was conducted in MEGA X [19], using the Maximum Likelihood (ML) method and General Time Reversible (GTR) model (discrete gamma distribution and invariable rate variation model) with a bootstrap of 500 replicates.

### 2.2. Characterization and Biotechnological Potential of Saccharopolyspora sp. NFXS83

#### 2.2.1. Production of Extracellular Lytic Enzymes

A colony of strain NFXS83 was picked into a 50 mL Falcon tube containing 10 mL of MB and incubated at 26 °C, 180 rpm for 3 days. After this period, 10 µL of the bacterial inoculum were directly inoculated (spots) onto marine basal solid media (3 g/L yeast extract, 5 g/L peptone and 15 g/L agar in natural seawater; pH 7.6) supplemented with different substrates (2 g/L; cellulose, chitin, starch, alginate, pectin, skimmed milk, respectively) (tributyrin, 10 mL/L; olive oil, 30 mL/L), in duplicate and incubated for 72 h at 26 °C. The enzymatic activities were determined on plates using Lugol’s Iodine Solution (prepared with 1 g I_2_ and 2 g KI in 300 mL of bi-distilled water), two days after inoculation. The degradation halos were measured in centimeters using the image processing tool ImageJ [20].

#### 2.2.2. Biosynthesis of Indole-3-Acetic Acid (IAA) and Other Indolic Compounds

Strain NFXS83 ability to produce IAA (indole-3-acetic acid), IPA (indole-3-propionic acid) and IBA (indole-3-butyric acid) was tested using the methodology based on the use of the Salkowski’s reagent described by Glickman and Dessaux [21]. The NFXS83 strain was pre-cultured in 10 mL of MB at 26 °C and 180 rpm shaking for 72 h, and posteriorly inoculated (10 µL) in a test tube containing 2.5 mL of MB supplemented with 0.5 g/L of Tryptophan (Sigma-Aldrich, Burghausen, Germany), in duplicate. The tubes were incubated at 26 °C and 180 rpm for 72 h. Culture samples of 1 mL were centrifuged at 7500× *g* rpm for 1 min and culture supernatants were recovered for analysis. To detect the indolic compounds, the supernatants were mixed (1:1 *v*/*v*) with the Salkowski reagent (4.5 g/L of FeCl_3_ in 10.8 M H_2_SO_4_) and added to polystyrene cuvettes. After a 5 min incubation period at room temperature and protected from light, the spectra between 400 and 600 nm was measured using an UV-Vis Spectrophotometer (Ultrospec 2100 pro, Biochrom, Holliston, MA, USA). The concentration of indolic compounds in the samples was determined based on the comparison with standard curves generated with known amounts of IAA, IPA, and IBA (range) that posteriorly received the Salkowski’s reagent as described above. The production of the three auxins tested was calculated based on the maximum absorbance wavelength of each of the compounds: 535 nm (IAA), 455 nm (IBA), and 460 nm (IPA) [21].

#### 2.2.3. Screening for Antimicrobial Activity

For the screening of the antimicrobial activity, strain NFXS83 was grown in 10 mL of Luria Broth medium (LB, 10 g/L tryptone, 5 g/L yeast extract and 5 g/L NaCl) at 30 °C and 150 rpm for 72 h. After this period, four spots of 10 µL of the NFXS83 bacterial solution were applied to LB agar plates and incubated for 72 h at 30 °C. A culture of *Staphylococcus aureus* ATCC 6538 grown overnight in LB medium was adjusted to an optical density at 600 nm (OD_600_) of 0.1 and streaked into the plates containing the NFXS83 strain grown in spots. After incubation at 30 °C for 24 h the halo of inhibition was measured. The screening was performed in duplicate.

#### 2.2.4. Microalgae (*Phaeodactylum tricornutum* CCAP 1055/1) Growth Promotion Assay

The microalga *Phaeodactylum tricornutum* CCAP 1055/1 was acquired from the Culture Collection of Algae and Protozoa (CCAP, Scotland, UK), and maintained axenic in MA plates. The microalga was cultivated in 2 L Schott flasks containing 1.2L of F/2 medium (seawater supplemented with 20 mL/L of Guillard’s -F/2- Marine Water Enrichment Solution) (Sigma-Aldrich, St. Louis, MO. USA), submitted to aeration of 0.2 L/min, at a temperature of 22 °C and in the presence of LED light at 70 µmol/s/m^2^ and a 16:8 h day/night cycle. The cultivation was carried out for 7 days. After the growth period, *P. tricornutum* cells were centrifuged at 3500× *g* rpm and 20 °C for 15 min and resuspended in F/2 medium.

The strain NFXS83 was grown in MB medium at 30 °C and 150 rpm for 72 h. The bacterial inoculum was centrifuged at 7500× *g* rpm and 4 °C for 8 min and the pellet resuspended in F/2 medium.

The microalga-bacteria co-cultivation assay was carried out in 6-well cell culture plates (VWR^®^, Leuven, Belgium), where 700 µL of the inoculum of *P. tricornutum* and NFXS83 were added to F/2 medium, for a total of 7 mL per well. The plates contained microalgae at a final concentration of 1 × 10^6^ cells/mL and a NFXS83 bacterial solution adjusted to a final OD_600_ = 0.1. The plates were incubated under LED lights, with a light intensity of 70 µmol/s/m^2^ on a 16:8 h light/dark cycle, with a temperature of 22 °C and agitation of 130 rpm. Samples were taken 5 and 10 days after inoculation and analyzed by flow cytometry (Muse^®^ Cell Analyzer, Luminex, Northbrook, IL, USA) and visualized under a microscope (Zeiss AX10, Kesselsdorf, Germany). Flow cytometry provided data on cell count, cell relative size by forward scattering (FSC), and cell auto-fluorescence (Red). A total of six replicates were conducted for each treatment (axenic *P. tricornutum*; axenic *P. tricornutum* + NFXS83 inoculation).

Furthermore, at the end of the assay, the fucoxanthin content of microalgae cells was quantified by analyzing methanol extracts of the culture samples by high-performance liquid chromatography (HPLC) as described by Wang et al. [22] with slight modifications. Briefly, 5 mL of culture samples were centrifuged (5000× *g*, 5 min), and the resulting pellets were resuspended in 5 mL of pure methanol, vortexed vigorously for 30 s and left in the dark for 24 h. The efficacy of the extraction was determined by confirming the pellets were completely white. Afterwards, the extracts were centrifuged to remove cell debris and directly used for HPLC analysis, which was performed Waters Alliance Separations Module e2695 (Waters, Dublin, Ireland) coupled with Photodiode Array Detector Module e2998 (HPLC-PDA). Separation of Fucoxanthin was achieved using a C18 reverse phase column (Phenomenex Luna 3u C18 (2) 100A 75´4.60mm) and a gradient elution at a constant flow rate of 1 mL/min with the following profile: 65% acetonitrile (ACN) and 35% milli-Q water (MQ) from 0 to 8 min, increasing until 90% ACN and 10% MQ from 8 to 11 min and maintained until 14 min, and then decreasing to 65% ACN and 35 % MQ from 14 to 20 min. The temperature of the column oven was 40 °C and the sample injection volume was 20 μL. The chromatogram was recorded using the PDA module at 445 nm. The quantification of fucoxanthin was performed by peak area integration and comparison to a calibration curve performed using fucoxanthin analytical standard (Sigma-Aldrich, St. Louis, MO, USA) prepared with pure methanol in the concentration range 0.05–0.6 ppm.

Statistical analyses were conducted by comparing means using the *t*-test function (Student’s *t*-test) in Microsoft Excel (Microsoft Corporation, Redmond, WA, USA). The differences were considered statistically significant when the *p* value was < 0.05.

### 2.3. Saccharopolyspora sp. NFXS83 Genome Sequencing and Analysis

The previously obtained NFXS83 DNA was used for genome sequencing, which was performed by Macrogen Inc. (Seoul, South Korea). The DNA library was constructed using the Illumina Nextera XT DNA Library Preparation Kit and was sequenced using the Illumina Novaseq6000 platform (2 × 150, paired end reads), generating a total of 11,088,684 reads. The obtained reads were trimmed using Trimmomatic [23] and using standard parameters (Sliding Window Trimming, window size 4, filter by quality, average quality of 25), leading to a total of 10,528,520 surviving reads which were used for the initial de novo genome assembly performed using Spades v.3.15.2 [24]. The assembly resulted in 70 contigs (>500 bp). The NCBI Prokaryotic Genome Annotation Pipeline [25] was used for strain NFXS83 genome annotation. The genome assembly can be found in the NCBI database under the accession number JAPFGB000000000.1.

The functional genome annotation was conducted using BlastKOALA [26] and BLASTp [27] searches against the UNIPROT database (2022_04) [28] performed in the Geneious Prime software [29]. Genes encoding carbohydrate active enzymes were predicted using the dbCAN2 webserver [30] and the tools DIAMOND (E-value < 1 × 10^−102^, coverage > 0.35) and HMMER (E-value < 1 × 10^−15^, coverage > 0.35). GH and other lytic protein domains were predicted using the InterProScan tool [31] which is also in the Geneious Prime software. Proteolytic enzymes were predicted using BLASTp searches against the MEROPS Peptidase Database [32] in the Geneious Prime software, for the conditions of E-value < 1 × 10^−15^ and coverage > 0.35. Secondary metabolite production genes/clusters were predicted using antiSMASH bacterial version v.6.0 [33] in relaxed mode. Phylogenomic analysis were conducted by calculating the Average Nucleotide Identity (ANI) and the Digital DNA-DNA Hybridization (DDH) values between *Saccharopolyspora* genomes (type strains) using OrthoANI [34].

## 3. Results and Discussion

### 3.1. 16S rRNA-Based Identification and Phylogenomic Analysis of Strain NFXS83

**Figure 1:**
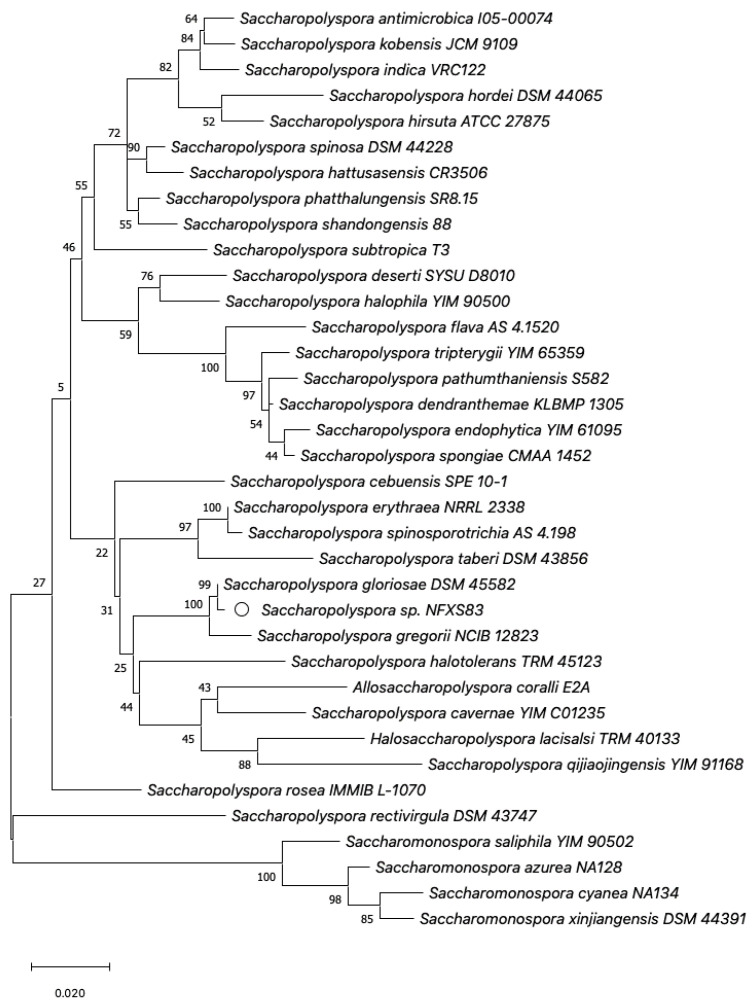
Phylogram based on the 16S rRNA gene of *Saccharopolyspora* type strains and other related type strains.

Phylogenetic analysis based on the 16S rRNA gene revealed that strain NFXS83 grouped in a cluster closer with *S. gloriosae* DSM 45582^T^ and *S. gregorii* NCIB 12823^T^. This cluster was found grouping next to the *S. hirsuta* (type species of the genus) group of strains (Figure 1). The *S. gloriosae* DSM 45582^T^ was the closest relative of strain NFXS83 (16S rRNA shared 99.86% identity); however, phylogenomic analysis revealed that strain NFXS83 does not belong to the *S. gloriosae* species. ANI analysis showed that the strain NFXS83 genome presents 93% identity to the *S. gloriosae* DSM 45582^T^ genome, a value that is below the 95% ANI threshold that delimits the same species [35]. In addition, a DDH estimate (GLM-based) of 52.90%, lower than the 70% DDH value used to delimit species [36], was obtained when comparing both genomes. Ultimately, the obtained results indicate that strain NFXS83 is a member of the currently described *Saccharopolyspora* genus, but does not belong to the *S. gloriosae* species, therefore will be further identified as *Saccharopolyspora* sp. NFXS83.

### 3.2. Characterization of Saccharopolyspora sp. NFXS83

#### 3.2.1. Production of Extracellular Lytic Enzymes in Marine Media

**Figure 2:**
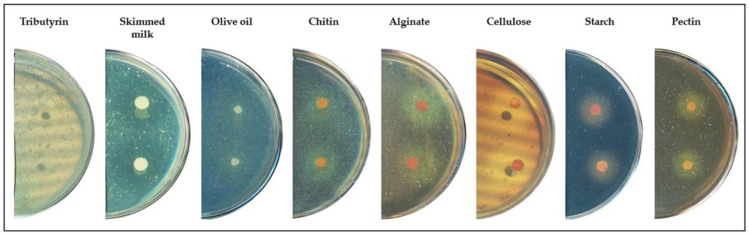
Lytic enzymatic activities observed for *Saccharopolyspora* sp. NFXS83 when cultivated in marine basal medium supplemented with different substrates. Enzymatic activities measured by the degradation halo diameter.

To assess the extracellular lytic enzymatic activities of *Saccharopolyspora* sp. NFXS83, an essay of degradation of different substrates added to a basal marine medium was performed. Degradation halos were detected in basal media supplemented with lipids (tributyrin and olive oil), proteins (skimmed milk) and carbohydrates (chitin, alginate, cellulase, starch, and pectin) (Figure 2). The size of the degradation halos was similar between the different substrates tested (~2 cm, 72 h after inoculation). Interestingly, the obtained results indicate that the enzymes produced by *Saccharopolyspora* sp. NFXS83 are functional and stable under high salt conditions (~3%, seawater NaCl concentrations), making them potential candidates for marine biotechnological applications. 

*Saccharopolyspora* strains are a known source of lytic enzymes, including several thermostable extracellular enzymes, such as β-galactosidase, alkaline phosphatase, α-amylase, and proteases of biotechnological interest [14]. For example, Chakraborty and colleagues [37] showed that the marine haloalkaliphilic *Saccharopolyspora* sp. A9 produced an extracellular α-amylase that was stable in the presence of wide range of NaCl concentrations and laboratory surfactants, detergents, and oxidants. The reported amylase showed novel properties that could lead to applications in detergent, food, and other industrial processes involving high salt concentrations.

#### 3.2.2. Synthesis of Indole-3-Acetic Acid (IAA) and other Indolic Compounds

*Saccharopolyspora* sp. NFXS83 presented the ability to synthesize IAA (6.25 ± 0.21 µg/mL), IBA (62.12 ± 6.35 µg/mL), and IPA (40.95 ± 3.89 µg/mL) from tryptophan, suggesting that this strain may influence auxin levels in marine environments/organisms. Previous studies have demonstrated that members of the *Saccharopolyspora* genus are able to produce IAA. For example, Gangwar and colleagues [38], showed that strains *Saccharopolyspora* sp. M13 and O9, isolated from medicinal plants in India, produced 11.1 µg/mL and 17.2 µg/mL of IAA, respectively. Moreover, many other soil and rhizosphere actinomycetes have also shown potential to produce IAA and promote plant growth [39,40]. As phytohormone-producing microorganisms, actinomycetes could also be potentially used to promote microalgal growth. Kumsiri et al. [41] showed that the actinomycete *Piscicocus intestinalis* WA3 could produce IAA as an algal growth promoting agent, leading to a significant increase in *Tetradesmus obliquus* AARL G022 biomass production, chlorophyll a content, and lipid productivity.

#### 3.2.3. Antimicrobial Activity of *Saccharopolyspora* sp. NFXS83

**Figure 3:**
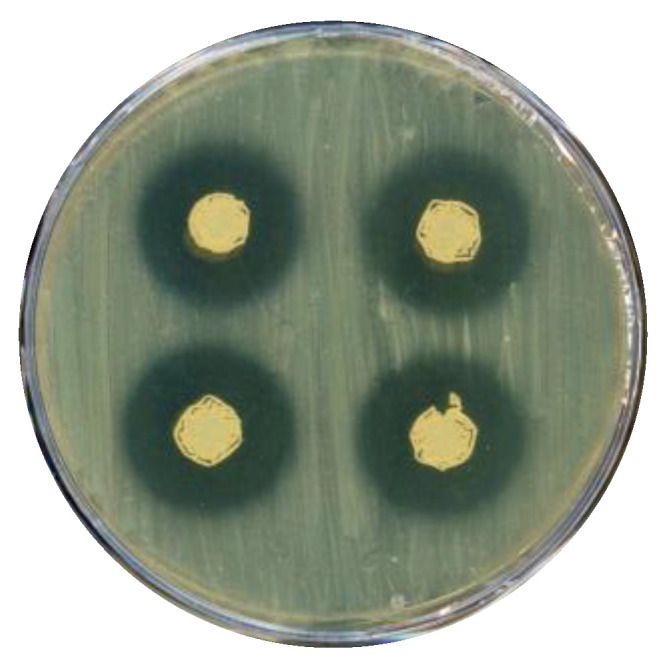
Antimicrobial activity of *Saccharopolyspora* sp. NFXS83 against *Staphylococcus aureus* ATCC 6538 in solid media. Halo measures ~1.39 cm.

Members of the *Saccharopolyspora* genus are known to synthesize a wide range of antimicrobial compounds [16]; therefore, strain NFXS83 was tested for its ability to inhibit the growth of the pathogen, *Staphylococcus aureus*. The antimicrobial activity of the strain NFXS83 against *S. aureus* was confirmed by the visualization of an inhibition zone surrounding the NFXS83 colony spots (Figure 3). These results indicate that strain NFXS83 produces diffusible secondary metabolites, such as antibiotics, capable of inhibiting the growth of *S. aureus.*

### 3.3. Saccharopolyspora sp. NFXS83 Promoted the Growth of Phaeodactylum tricornutum CCAP 1055/1

**Figure 4:**
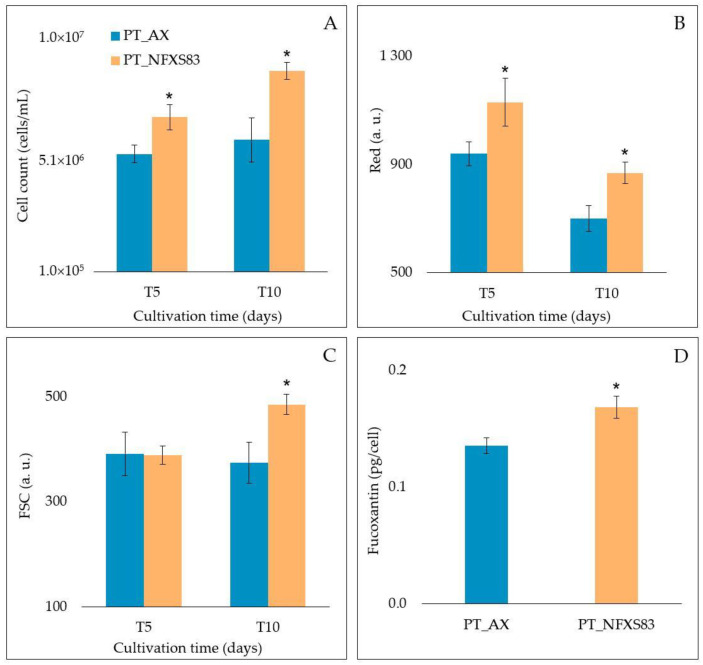
Growth dynamics of *Phaeodactylum tricornutum* cultivated under axenic conditions (PT_AX) and in co-cultivation with *Saccharopolyspora* sp. NFXS83 (PT_ NFXS83). (**A**) *P. tricornutum* cell count (cells/mL); (**B**) auto-fluorescence (Red); (**C**) front scatter (FSC); (**D**) fucoxanthin content (pg/cell) for T10. Values are average of *n* = 6 ± standard deviations. * Statistically significant (*p* < 0.05).

The co-cultivation assay showed that the inoculation of *P. tricornutum* CCAP 1055/1 with *Saccharopolyspora* sp. NFXS83 led to an increase of the microalgae cell count, red fluorescence, size, and fucoxanthin accumulation when compared to the microalgae cultivated under axenic conditions (Figure 4A–D).

The presence of *Saccharopolyspora* sp. NFXS83 led to a significant increase (31%) in the microalgae cell number, and this effect was already observed 5 days after the initial inoculation. The effect maintained leading to a 51% increase observed at 10 days after inoculation (Figure 4A). In the same period, the increase of red fluorescence (Figure 4B) and cell size (Figure 4C), 24% and 30%, respectively, was also significant. The amount of fucoxantin accumulated per cell was also calculated, leading to an increase of 24% of this content when compared to the microalgae cultivated in axenic conditions (Figure 4D).

**Figure 5:**
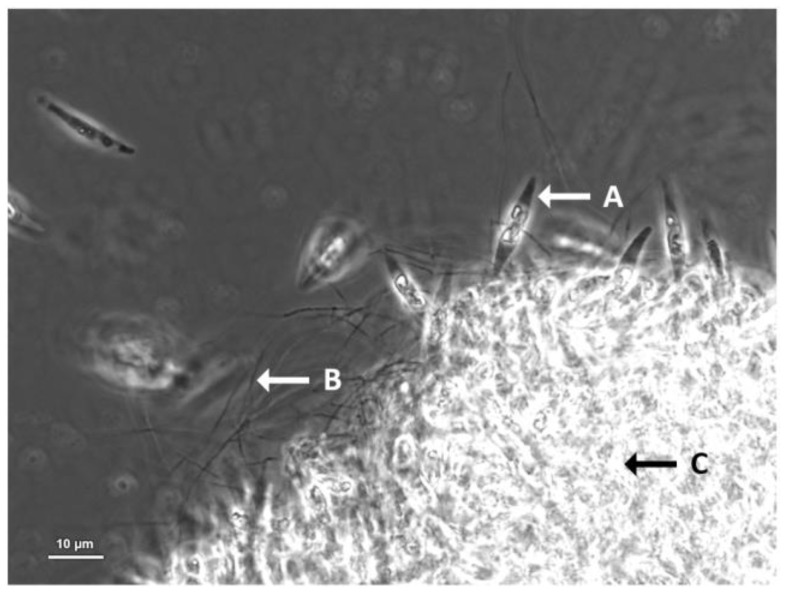
*Saccharopolyspora* sp. NFXS83 co-cultured with *Phaeodactylum tricornutum* CCAP 1055/1, 10 days after inoculation, under ×1000 amplification. (**A**) *P. tricornutum* free-living cell; (**B**) Hypha-like growth of *Saccharopolyspora* sp. NFXS83; (**C**) *P. tricornutum* biofilm with *Saccharopolyspora* sp. NFXS83.

Microscope observations (Figure 5) showed that the NFXS83 bacterium promoted the aggregation of *P. tricornutum* cells and the formation of films, which may facilitate the possible exchanges of compounds that may occur between the microalga and the bacterium.

Several studies have showed the beneficial impacts of bacteria in the development of several microalgae [42], including *P. tricornutum* [43,44]. Bacteria thrive in the microalgae phycosphere; they feed on algal exudates while also producing biostimulant compounds, such as IAA, and recycling nutrients [42,45]. The previously described results shown the ability of the strain NFXS83 to produce different auxins (IAA, IBA and IPA), which could be an explanation for the observed *P. tricornutum* growth improvement. In fact, several reports have shown that bacterial-synthesized IAA positively impacted the growth of several distinct microalgae [46,47]. Nevertheless, other factors may be involved in the beneficial interaction between *Saccharopolyspora* sp. NFXS83 and *P. tricornutum*, and new studies are necessary to unveil the detailed molecular interactions between these organisms.

### 3.4. General Properties of Saccharopolyspora sp. NFXS83 Genome

The genome of *Saccharopolyspora* sp. NFXS83 is 6.896 Mbp in length with an average GC content of 71%. A total of 5999 genes were predicted, of which 5836 correspond to complete protein-coding sequences (CDSs). A total of 55 RNA-related genes were also found. The analysis performed using BlastKOALA resulted in the functional annotation of 2446 from a total of 5836 CDSs (41.9%) (Table S1). The annotated CDS were mostly involved in carbohydrate metabolism (301), genetic information processing (443), and signaling and cellular processes (257), followed by amino acid metabolism (219), environmental information processing (175), and the metabolism of cofactors and vitamins (125), energy (108), nucleotides (100), and lipids (91).

### 3.5. Genomic Insights into Saccharopolyspora sp. NFXS83 Lytic Enzymes

#### 3.5.1. Glycosyl Hydrolases and Pectate Lyase Family Enzymes

Strain NFXS83 presented the ability to degrade extracellular chitin, starch, cellulose, pectin, and alginate, indicating the presence of extracellular lytic enzymes in its genome. In fact, a total of 60 CDSs encoding Glycosyl Hydrolase (GH) enzymes, divided into 33 different GH families, were detected in the genome of *Saccharopolyspora* sp. NFXS83. From these, 34 GHs contained signal peptides, including chitinases, glucanases, rhamnosidases, and several other lytic enzymes (Table S2). Only two CDS encoding enzymes from the Polysaccharide Lyase (PL) family were found, both containing signal peptides.

Chitinases (GH18) were amongst the most prevalent signalP+ GHs in the strain NFXS83 genome. The encoded four chitinases were similar (33–73%) to *Saccharopolyspora erythraea* extracellular chitinase [48] (Table S3). Moreover, the NFXS83 genome contained one signalP+ beta-N-acetylhexosaminidase (GH20, OOZ19_22990) which presented 44% identity to the described beta-N-acetylhexosaminidase of *Cellumonas firmi* involved in the degradation of beta-N-acetylglucosaminides and N-acetylchitooligomers that result from chitin degradation processes [49] (Table S3). In addition, NFXS83 also contained several deacetylases, possibly involved in chitin deacetylation to chitosan, and one gene (OOZ19_20955) encoding a signalP+ chitosanase with high identity (64.2%) to a *Streptomyces* sp. N174 chitosanase [38] (Table S3), further suggesting that strain NFXS83 may also degrade chitin via chitosan.

Three genes encoding GH13 family enzymes (amylase) were found, however, only one gene (OOZ19_20745) encoding an amylase domain-containing protein presented a signal peptide region (Table S3). Furthermore, one other SignalP+ CDS (OOZ19_03275) from the GH97 family identical (36.0%) to a Glucan 1,4-alpha-glucosidase SusB from *Bacteroides thetaiotaomicron* was annotated. This glucoamylase presents the ability to hydrolase the alpha-1,4-, alpha-1,6-, alpha-1,3- and alpha-1,2-glucosidic linkages during starch degradation [50].

As for cellulose degradation, despite the absence of genes encoding typical cellulose hydrolyzing enzymes (endo-β-1,4-glucanase or exo-1,4-glucanase activity) in the *Saccharopolyspora* sp. NFXS83 genome, several genes encoding other glucanases and glucosidases were detected (Table S3). Five of these genes encoded signalP+ enzymes (GH64, OOZ19_03725; GH55, OOZ19_09715; GH30, OOZ19_18355; and GH16, OOZ19_28405) and are possibly involved in the demonstrated cellulolytic activities of strain NFXS83.

Eight genes encoding enzymes capable of hydrolyzing different components of pectin were also found: two endo-alpha-(1>5)-L-arabinanases, one beta-L-arabinobiosidase, one alpha-galactosidase, three beta-galactosidases, and one rhamnogalacturonan endolyase (Table S3). The signalP+ containing protein encoded by OOZ19_04700, presented high identity (54.7%) to *Bacillus subtillis* 168 rhamnogalacturonan endolyase YesW, whose ability to hydrolyze rhamnogalacturonan (main component of pectin) has already been demonstrated [39]. Additionally, a CDS (OOZ19_16415) encoding a signalP+ enzyme from the PL14 family (alginate lyase activity) was detected in the NFXS83 genome (Table S2).

Interestingly, the genome of *Saccharopolyspora* sp. NFXS83 also contained three genes encoding lysozymes, known for their ability to lyse bacterial cell membranes [40]. Of these, only one was signalP+ (OOZ19_20845), which may be indicative of extracellular activity (Table S3).

From the 62 CDS presented in Table S2, 12 did not present similarity to any of the entries found in the UniProt database, suggesting that *Saccharopolyspora* sp. NFXS83 encodes new protein coding sequences not previously identified from the GH23, GH33, GH43, GH87, GH93, GH114, GH172, and PL14 families. Thus, this isolate could be a source of novel enzymes adapted to marine environments with strong potential for biotechnological applications.

#### 3.5.2. Lipases, Esterases and Proteases

The degradation of proteins and lipids in marine media was also determined for strain NFXS83. This is consistent with the presence of 12 lipases and 37 esterase-encoding genes in its genome. From the 12 genes encoding lipases, 3 encoded proteins that contained signal peptides and extracellular domains (Table S4). Two CDS (OOZ19_14095 and OOZ19_09905) presented 40.4% and 25.5% similarity, respectively, to the Lipase 2 from *Streptomyces coelicolor*, and one (OOZ19_29350) presented 31.2% similarity to Lipase EstA from *B. subtillis*. Both Lipase 2 and Lipase EstA have been shown to exhibit high lipolytic activities [51,52]. Regarding esterases, none of the 37 genes encoding these enzymes contained signal peptides.

BLAST analysis against the MEROPS database led to the prediction of 150 genes encoding enzymes belonging to different peptidase families (Table S5), of which 40 contained signal peptide. Amongst these were several CDS encoding serine proteases (Table S6), one of which (OOZ19_12730) presented high identity (43.1%) to a homolog Alkine serine protease from *Lecanicillium psalliotae*, an extracellular enzyme previously studied for its ability to degrade a wide range of substrates, including casein and gelatin [53].

### 3.6. Saccharopolyspora sp. NFXS83 Presents a Wide Range of Gene Clusters Involved in Secondary Metabolite Production

The analysis conducted using ANTISMASH revealed the presence of multiple biosynthetic gene clusters (BGCs) involved in secondary metabolite production, including polyketides, non-ribosomal peptides, lanthipeptides, bacteriocins or other unspecified ribosomally synthesized and post-translationally modified peptide products (RiPP-like), non-alpha poly-amino acids, such as e-Polylysin (NAPAA), ectoine, and terpenes, in the genome sequence of *Saccharopolyspora* sp. NFXS83 (Table 1).

Interestingly, several of these clusters are involved in the production of antimicrobial compounds, such as erythreapeptin, kiamycin, and ε-Poly-L-lysine, further corroborating the observed antibacterial effects of strain NFXS83. The antimicrobial gene clusters are analyzed in detail below.

Other secondary metabolites gene clusters were also detected, such as those involved in terpene production, including geosmin, which is a known volatile of actinomycetes [54,55], and an unknown carotenoid pigment (isorenieratene-like), as well as a gene cluster containing the ectoine production genes. These secondary metabolites may play key important roles in the ecological adaptations of *Saccharopolyspora* sp. NFXS83. For example, geosmin is a chemical signaling compound created by toxin-producing microbes to deter predation by eukaryotic organisms [55]. Ectoine is a known osmolyte that plays a key role in the osmotic stress tolerance of several microorganisms [56].

#### 3.6.1. BGC 1

A hybrid cluster containing homologs of the kirromycin, rimosamide, bottromycin A2/D, and cyanobactin biosynthesis genes was detected in the NFXS83 genome (Table 2). Comparative analysis revealed that cluster 1 contained high identity homologs of the *Streptomyces collinus* Tu365 *kir* genes [57] (Table 2), suggesting the production of a kirromycin-like narrow-spectrum antibiotic. Kirromycin binds to prokaryotic elongation factor (EF) Tu, resulting in the inhibition of protein biosynthesis [58]. Next to the *kir* genes, a Non-Ribosomal Peptide Synthase (NRPS) presenting homology to the *Streptomyces rimosus subsp. rimosus* ATCC 10970 *rmoI* gene involved in rimosamide biosynthesis was also detected. The rimosamides and detoxins family compounds exhibit anti-antibiotic activity, which may serve as defense mechanisms to resist specific actinobacteria antibiotics [59]. The presence of a *rmoI*-like gene in the vicinity of strain NFXS83 kirromycin-like antibiotic biosynthesis genes suggests that the *rmoI*-like produced compound may act as an “antidote” to the kyrromicin-like antibiotic.

The bottromycin gene cluster of *Saccharopolyspora* sp. NFXS83 (OOZ19_02920-02965) presented high identity (from 58.2 to 78% identity) to the bottromycin A2 biosynthetic gene cluster from *Streptomyces scabiei* 87.22 [60] (Table 2). Bottromycins are a class of macrocyclic peptides that present potent antibacterial activity, even against multidrug-resistant human and plant pathogens, such as *Xanthomonas oryzae* [61]. Homologs of the cyanobactin biosynthesis genes were also detected in the vicinity of the bottromycins production cluster. These included two biosynthetic genes presenting similarity to the Kawaguchipeptin A and Limnothamide biosynthesis genes of *Microcystis aeruginosa* NIES-88 and *Limnothrix* sp. CACIAM 69d, respectively. Interestingly, BLASTp analysis revealed that homologs of the cyanobactin-like encoding genes of *Saccharopolyspora* sp. NFXS83 (OOZ19_02990 and OOZ19_03000) were not detected in other *Saccharopolyspora* spp. genomes, suggesting that this may be a strain-specific characteristic. Cyanobactin-like peptides are known to exhibit antibacterial effects as well as allelopathy against cyanobacteria and small grazing organisms [62,63,64]. It is possible that *Saccharopolyspora* sp. NFXS83 cyanobactin-like genes may play a role in its marine environment colonization activities, where it interacts with other marine microbes, including cyanobacteria and other members of phyto and zooplankton.

#### 3.6.2. BGCs 2, 3, 4, 7, 8, 10, 11

A cluster (BGC2) containing a *gfsA* gene homolog was found in the NFXS83 genome (Table 3). The *gfsA* gene is part of a biosynthetic gene cluster of *Streptomyces graminofaciens* encoding FD-891, a 16-membered macrolide antibiotic with antitumoral effects [65]. While some similarity was found between *gfsA* and NFXS83 OOZ19_03940 encoded proteins the remaining *gfsBCDEF* genes were not detected in the NFXS83 genome. These results suggest that NFXS83 OOZ19_03940 may encode a different type of antibiotic compound.

The BGC3 of *Saccharopolyspora* sp. NFXS83 presented high similarity to the *Streptomyces griseus* polycyclic tetramate macrolactams (PTMs) gene cluster [66] (Table 3). PTMs from a wide range of microorganisms are known to present antifungal, antibiotic, and antioxidant properties [66].

The NFXS83 BGC4 identified throughout AntiSMASH analysis (Table 3), contained a homolog (OOZ19_07305) of the ε-Poly-L-lysine synthase gene of *Epichloe festucae* [67], suggesting that *Saccharopolyspora* sp. NFXS83 also synthesizes an ε-Poly-L-lysine-like compound that is known for its antimicrobial activity; it is widely used in food, pharmaceutical, and medical applications [68].

An althiomycin-like BGC (BGC 7) (Table 3) that contains homologs of the *Myxococcus xanthus* DK897 *almAB* and *almF* [69] was detected in the NFXS83 genome. Althyomycin is a broad-spectrum sulfur-containing antibiotic mainly synthesized by *Streptomyces*, soil Myxobacteria and entomopathogenic *Serratia* [70].

A T1PKS containing cluster (BGC8) harboring homologs of the *Streptomyces cyanogenus* lucensomycin BGC [71] was also found in *Saccharopolyspora* sp. NFXS83 genomic repertoire (Table 3). Lucensomycn is a macrolide presenting antifungal activity used in food and agricultural applications [72].

Finally, two lanthipeptide encoding BGCs (10,11) were found in the NFXS83 genome (Table 3), and these contained the genes involved in erythreapeptin and kyamicin biosynthesis, respectively. Both compounds are known products of *Saccharopolyspora* strains [73,74]; however, their modes of action remain elusive.

### 3.7. Genomic Insights into the Microalgae-Growth Promoting Properties of Saccharopolyspora sp. NFXS83

Genomic analysis revealed the presence of multiple genes that could be involved in the beneficial interactions between *Saccharopolyspora* sp. NFXS83 and microalgae. Genes involved in the production of IAA were detected in the NFXS83 genome, such as several aminotransferases responsible for the conversion of tryptophan to indolepyruvic acid (IPyA), a YUCCA-like enzyme (OOZ19_04645, 31.8% identity to *Arabidopsis* YUC3 gene) which converts IPyA to IAA [75], and an homolog (OOZ19_04915) of the flavin-dependent L-tryptophan oxidase RebO (52.9% identity to RebO from *Lentzea aerocolonigenes*) involved in the transformation of tryptophan to 2-iminio-3-(indol-3-yl)propanoic acid, a possible precursor of indole-3-propionic acid (3-(3-Indolyl)propanoic acid) (IPA). Moreover, *Saccharopolyspora* sp. NFXS83 contains the genetic machinery involved in the production of several vitamins and co-factors, including pantothenate (vitamin B5), biotin (vitamin B7), tetrahydrofolate (vitamin B9), and cobalamin (vitamin B12) (Table S7). Several studies have revealed the importance of bacterial synthesized vitamins in microalgae growth [76,77,78].

## 4. Conclusions

This study provides an in-depth analysis of *Saccharopolyspora* sp. NFXS83 and its biotechnological potential. The strain produced functional and stable extracellular lytic enzymes under high salt conditions, presented antimicrobial activity and promoted the growth and pigment accumulation of *Phaeodactylum tricornutum*. The genomic analysis of the strain revealed the presence of several unique genes involved in lytic enzyme production, various gene clusters involved in the production of secondary metabolites (e.g., antimicrobial compounds, terpenes, and carotenoids) of great interest, as wells as genes involved in the biosynthesis of microalgae growth promoting substances, further reinforcing the marine biotechnological potential of this strain. Overall, this study lays the foundation for future studies exploring *Saccharopolyspora* sp. NFXS83 applications in various marine biotechnological processes.

## Figures and Tables

**Table 1:** Secondary metabolite gene clusters identified in the genome sequence of *Saccharopolyspora* sp. NFXS83. Only clusters with >50% similarity to known clusters are showed.

BGC Number	Cluster Type	Most Similar Known Cluster	Similarity
1	NRPS, transAT-PKS, T1PKS, bottromycin, cyanobactin	Kirromycin biosynthetic gene cluster from *Streptomyces collinus Tu 365*	57%
2	T1PKS	FD-891 biosynthetic gene cluster from *Streptomyces graminofaciens*	50%
3	NRPS, T1PKS	SGR PTMs biosynthetic gene cluster from *Streptomyces griseus subsp. griseus* NBRC 13350	66%
4	NAPAA	ε-Poly-L-lysine biosynthetic gene cluster from *Epichloe festucae*	100%
5	Terpene	Geosmin biosynthetic gene cluster from *Streptomyces coelicolor* A3(2)	100%
6	Terpene	Isorenieratene biosynthetic gene cluster from *Streptomyces griseus subsp. griseus* NBRC 13350	57%
7	NRPS-like, NRPS, T1PKS	Althiomycin biosynthetic gene cluster from *Myxococcus xanthus*	100%
8	T1PKS	Lucensomycin biosynthetic gene cluster from *Streptomyces cyanogenus*	68%
9	Ectoine	Ectoine biosynthetic gene cluster from *Streptomyces anulatus*	100%
10	RRE-containing, lanthipeptide-class-III	Ery-9 biosynthetic gene cluster from *Saccharopolyspora erythraea* NRRL 2338	100%
11	Lanthipeptide-class-II	Kyamicin biosynthetic gene cluster from *Saccharopolyspora* sp.	100%

NRPS—Non-ribosomal peptide synthetase; **TIPKS**—Type I Polyketide synthase; **transAT-PKS**—Trans-AT Polyketide synthase; **NAPAA**—Non-alpha poly-amino acids; **RRE-containing**—RRE-element containing cluster; **Lanthipeptide class II**—Class II lanthipeptides, such as mutacin II (U40620); **Lanthipeptide class III**—Class III lanthipeptides, such as labyrinthopeptin (FN178622).

**Table 2:** Detailed analysis of *Saccharopolyspora* sp. NFXS83 hybrid biosynthetic gene cluster 1.

Type	Locus Tag	Mibig Cluster	Mibig Protein ID	ID %	Coverage %	Cluster, Host
NRPS	OOZ19_02780	BGC0001760	WP_004571777.1 (RmoI)	57.0	107.8	Rimosamide, *Streptomyces rimosus* subsp. *rimosus* ATCC 10970
NRPS	OOZ19_02890	BGC0001070	CAN89633.1 (KirAIII)	61.0	102.0	Kirromycin, *Streptomyces collinus* Tu 365
T1PKS	OOZ19_02875		CAN89636.1 (KirAVI)	56.0	101.4	
NRPS	OOZ19_02865		CAN89638.1 (KirB)	64.0	99.0	
RIPP-like	OOZ19_02920	BGC0001157	CBG72695.1	70	100.5	Bottromycin A2, *Streptomyces scabiei* 87.22
	OOZ19_02925		CBG72688.1	67	96.4	
	OOZ19_02930		CBG72689.1	62	102.6	
	OOZ19_02935		CBG72690.1	65.0	92.8	
	OOZ19_02940		CBG72691.1	70.0	100.2	
	OOZ19_02945		CBG72692.1	65.0	92.1	
	OOZ19_02950		CBG72693.1	56.0	98.7	
	OOZ19_02955		CBG72694.1	85.0	89.1	
	OOZ19_02960		CBG72695.1	72.0	99.1	
	OOZ19_02965		CBG72696.1	63.0	99.6	
RIPP-like	OOZ19_02990	BGC0001632	KXS89937.1	40.0	105.9	Kawaguchipeptin A, *Microcystis aeruginosa* NIES-88
RIPP-like	OOZ19_03000	BGC0002629	RFP52076.1	41.0	100.6	Limnothamide biosynthetic gene cluster, *Limnothrix* sp. CACIAM 69d

**Table 3:** Detailed analysis of *Saccharopolyspora* sp. NFXS83 biosynthetic gene clusters 2, 3, 4, 7, 8, 10, 11.

Type	Locus Tag	Mibig Cluster	Mibig Protein ID	ID %	Coverage %	Cluster, Host
T1PKS	OOZ19_03940	BGC0000058	BAJ16467.1 (GFSA)	46.0	102.4	FD-891, *Streptomyces graminofaciens*
NRPS, T1PKS	OOZ19_04725	BGC0001046	BAG17643.1 (SGR_814)	64.0	102.4	SGR PTM, *Streptomyces griseus*
	OOZ19_04730		BAG17642.1 (SGR_813)	63.0	96.7	
	OOZ19_04735		BAG17641.1 (SGR_812)	77.0	98.2	
	OOZ19_04740		BAG17640.1 (SGR_811)	66.0	100.0	
NAPAA	OOZ19_07305	BGC0002174	BBU42014.1 (EPLS)	48.0	97.4	ε-Poly-L-lysine, *Epichloe festucae*
NRPS, T1PKS	OOZ19_10385	BGC0000955	CCA29202.1 (AlmA)	49.0	101.9	Althiomycin, *Myxococcus xanthus*
	OOZ19_10380		CCA29203.1 (AlmB)	51.0	102.5	
	OOZ19_10375		CCA29204.1 (AlmF)	53.0	97.3	
T1PKS	OOZ19_12435	BGC0002333	QSE03591.1 (LcmC)	62.0	100.7	Lucensomycin, *Streptomyces cyanogenus*
	OOZ19_12440		QSE03601.1 (LcmB)	49.0	100.5	
	OOZ19_12445		QSE03603.1 (LcmE)	55.0	103.8	
RRE-containing, lanthipeptide-class-III	OOZ19_26865	BGC0000513	CAM03499.1	72.0	99.2	Ery-9, *Saccharopolyspora erythraea* NRRL 2338
	OOZ19_26870		CAM03500.1	67.0	102.1	
	OOZ19_26875		CAM03501.1	100.0	100.0	
	OOZ19_26880		CAM03502.1	69.0	99.6	
Lanthipeptide-class-III	OOZ19_28805	BGC0002346	QDF63352.1 (KyaR1)	68.0	94.8	Kyamicin, *Saccharopolyspora* sp.
	OOZ19_28810		QDF63353.1 (Kyaorf11)	77.0	91.5	
	OOZ19_28815		QDF63354.1 (KyaL)	73.0	99.6	
	OOZ19_28820		QDF63355.1 (KyaK)	71.0	99.7	
	OOZ19_28825		QDF63356.1 (KyaR)	84.0	99.5	
	OOZ19_28830		QDF63357.1 (KyaN)	72.0	100.0	
	OOZ19_28835		QDF63358.1 (KyaA)	78.0	100	
	OOZ19_28840		QDF63359.1 (KyaM)	74.0	99.7	
	OOZ19_28845		QDF63360.1 (KyaX)	78.0	100.3	
	OOZ19_28850		QDF63361.1 (KyaT)	90.0	99.7	
	OOZ19_28855		QDF63362.1 (KyaH)	82.0	100.0	

## Data Availability

The genome of strain NFXS83 is available in the NCBI database under the accession number JAPFGB000000000.1.

## Supplementary Materials

The following supporting information can be downloaded at: https://www.mdpi.com/article/10.3390/microorganisms11040902/s1, Table S1: BlastKOALA functional annotation of the NFXS83 genome; Table S2: Results of the carbohydrate active enzymes predicted using dbCAN; Table S3: Genes involved in carbohydrate metabolism, predicted with BLASTp against the UNIPROT database; Table S4: Genes encoding signalP+ enzymes involved in extracellular degradation of lipids, predicted with BlastKOALA; Table S5: Genes encoding signalP+ enzymes involved in extracellular degradation of proteins, predicted using BLASTp against the MEROPS Peptidase Database; Table S6: Genes encoding signalP+ enzymes involved in extracellular degradation of proteins, predicted using BLASTp against the MEROPS Peptidase Database and identified with the UniProt database; Table S7: Genes involved in metabolism of cofactors and vitamins.
